# Correlation of ultrasound bladder vibrometry assessment of bladder compliance with urodynamic study results

**DOI:** 10.1371/journal.pone.0179598

**Published:** 2017-06-16

**Authors:** Mahdi Bayat, Viksit Kumar, Max Denis, Jeremy Webb, Adriana Gregory, Mohammad Mehrmohammadi, Mathew Cheong, Douglas Husmann, Lance Mynderse, Azra Alizad, Mostafa Fatemi

**Affiliations:** 1 Department of Physiology and Biomedical Engineering, Mayo Clinic, Rochester, MN, United States of America; 2 Department of Radiology, Mayo Clinic, Rochester, MN, United States of America; 3 Department of Urology, Mayo Clinic, Rochester, MN, United States of America; Tianjin University, CHINA

## Abstract

**Purpose or objective:**

The objective of this study is to assess correlation between bladder wall mechanical properties obtained by ultrasound bladder vibrometry (UBV) and urodynamic study (UDS) measurements in a group of patients undergoing clinical UDS procedure.

**Materials and methods:**

Concurrent UBV and UDS were performed on 70 patients with neurogenic bladders (56 male and 14 female). Bladder wall mechanical properties measured by UBV at different filling volumes were correlated with recorded detrusor pressure (*Pdet*) values. Mean, median and standard deviation of correlation values were calculated and the significance of these observations was tested.

**Results:**

Bladder wall mechanical properties obtained by UBV as group velocity squared and elasticity showed high correlations with *Pdet* measured at different volumes (median correlation 0.73, CI: 0.64–0.80 and 0.72, CI: 0.56–0.82 respectively). The correlation of group velocity squared and elasticity with *Pdet* were both significantly higher than 0.5.

**Conclusions:**

The results of this study suggest that UBV can closely monitor changes in bladder wall mechanical properties at different volumes in a group of patients undergoing UDS. The high correlation between UBV parameters and detrusor pressure measurements suggests that UBV can be utilized as a reliable and cost-effective tool for assessment of the bladder wall mechanical changes in a noninvasive fashion.

## Introduction

Proper functionality of the human urinary system highly depends on bladder compliance. The heterogeneous combination of a smooth muscle layer and fibrous connective tissues provides a stretching capability which is essential for unidirectional flow of the urine from renal tracks, adequate storage of the urine and activation of the neural system for volunteer voiding [1]. A number of physiological and neurological abnormalities can deteriorate this functionality leading to a noncompliant bladder [2–5]. In case of neurological defects such as spinal cord injuries, excessive growth of the bladder fibrotic tissue can increase its rigidity, which in turn can lead to a noncompliant bladder [6]. The changes on the bladder’s mechanical properties occur gradually over the course of several months [7]; therefore, patients in risk of developing bladder non-compliance are routinely examined to detect these changes in an early stage and avoid the progression by prescribing appropriate medication.

Currently, the urodynamic study (UDS) is the clinical routine for such examination. One of the main components of UDS is the study of bladder detrusor compliance via gradual filling of the bladder through an intravesical catheter and simultaneous measurement of the detrusor pressure (*Pdet*) via two pressure sensing catheters. By analyzing the results of UDS, physicians are able to observe the bladder’s physiological and neurological response to excess fluid at different volumes. Information such as leak points, bladder overactivity, and bladder compliance (i.e., the maximum volume change over the maximum detrusor pressure change) can be immediately derived from such analysis [8]. The accuracy of this method can well justify its invasiveness and high cost, however, a more versatile, inexpensive and reliable technique with less invasive nature is desired.

Among other factors, the cystometry analysis performed by UDS evaluates the bladder mechanical properties based on a pressure response to filling fluid volume. A number of studies have considered other properties of the bladder wall which may be indirectly related to the bladder wall mechanical properties. For example, noninvasive techniques based on ultrasound measurement of the bladder weight and wall thickness have been suggested for evaluation of the bladder physiological and neurological state [9–12]. However, the review in [13] concludes that these methods have not shown sufficient clinical relevance to be able to completely or partially replace common UDS examination. Hence, a noninvasive method which can directly measure the bladder mechanical properties under different loading conditions can be highly valuable.

Ultrasound elastography methods have emerged as noninvasive tools for assessment of the tissue mechanical properties in the recent years. Shear wave elastography, in particular, has gained interest in assessing tissue stiffness (elasticity) as a potential biomarker for predicting different diseases and pathological conditions such as breast cancer and malignant thyroid nodules [14–16]. In this method, an ultrasonic tone burst is spatially focused at a point inside tissue by using an array transducer. Tissue attenuation and reflection creates a localized body force which in turn launches transient shear waves that move away from the focal region. By point-wise measurement of the speed of these waves (via high speed imaging), it is possible to obtain a measure of the localized tissue stiffness in terms of shear or Young’s modulus. However, adopting this method for assessment of the elasticity in layered structures such as bladder poses a number of challenges. A previous study has shown that selection of an appropriate model for analysis of the transient waves along the bladder wall is essential for obtaining a true measure of elasticity. Ultrasound bladder vibrometry (UBV) utilizes acoustic radiation force to excite transient waves in tissues. UBV uses a Lamb wave model to extract parameters of elasticity and viscosity based on standard least-square fitting procedures and wave dispersion analysis along the bladder wall. Lamb waves are a family of mechanical waves that propagate along thin viscoelastic plates. The results of UBV are reported in terms of bladder wall’s modulus of elasticity as well as Lamb wave group velocity. The modulus of elasticity is obtained from the analysis of wave phase velocity dispersion while group velocity is measured as the speed of the wave maximum amplitude along the bladder wall [17]. Preliminary results of this method have shown that both elasticity and Lamb wave group velocity increase at higher volumes [18, 19]. The rate of this increase, however, depends on the bladder compliance such that for noncompliant bladder a rapid change is expected while a compliant normal bladder mostly shows a gradual increase [19].

In this study, we present the results of UBV in a group of patients undergoing UDS. The aim of this study is to establish the relationship between UBV measurements of the bladder mechanical properties and UDS outcomes in terms of detrusor pressures at different filling volumes. We use statistical analysis tools to show the strength of this relationship and discuss the utility of UBV as a novel, noninvasive and versatile technique for fast and accurate assessment of the bladder wall mechanical state.

## Material and method

### Ultrasound bladder vibrometry

**Fig 1:**
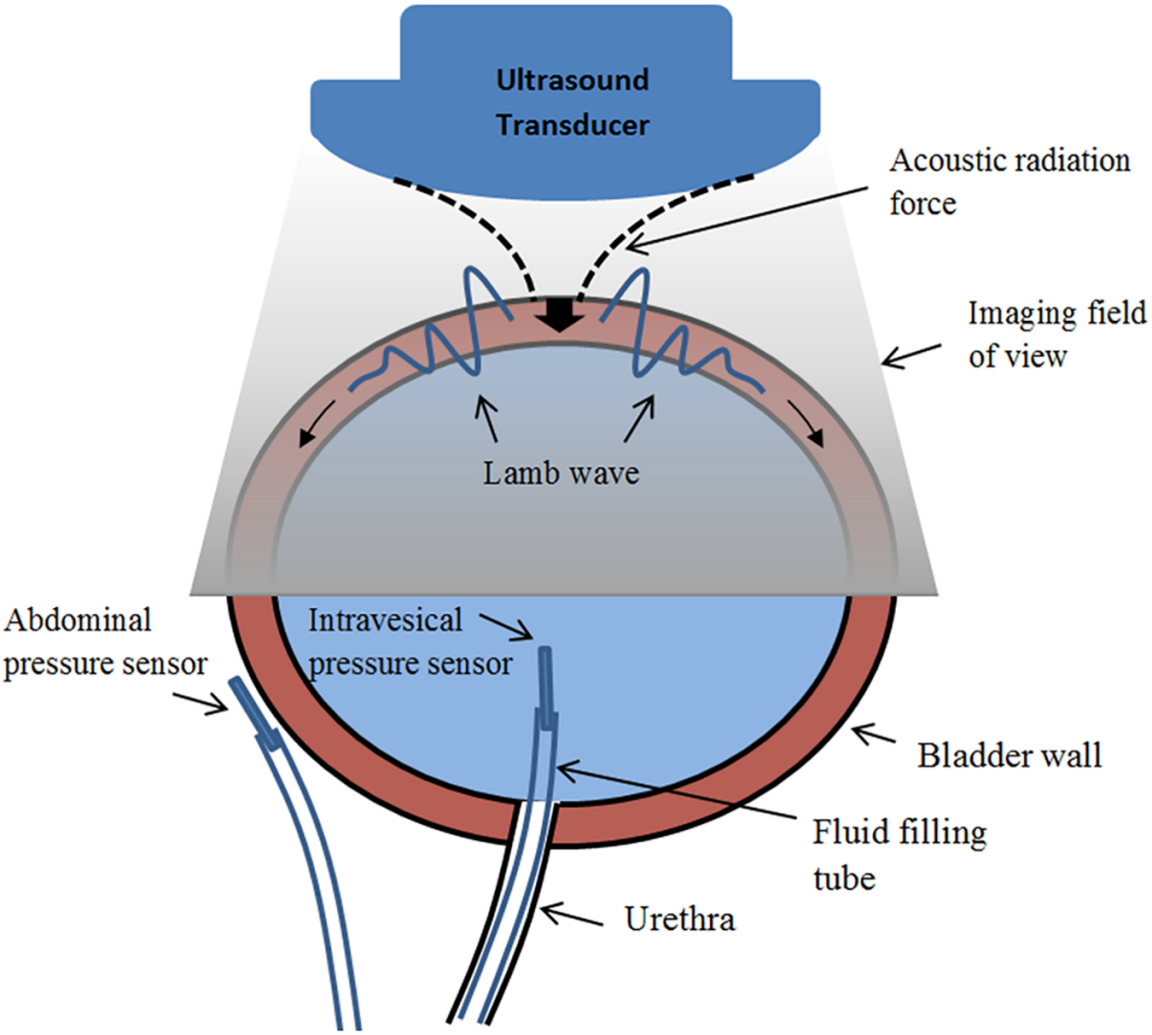
Concurrent UDS and UBV measurement setup for studying bladder compliance.

Human bladder can be modelled as an expandable viscoelastic thin shell immersed in and filled with incompressible fluid [19]. Mechanical excitation of such shell creates mechanical waves that propagate along the bladder wall at a speed that is a function of tissue geometry and viscoelastic properties. A Lamb wave representation has shown to closely model these waves [19]. In UBV, using acoustic radiation, a localized force is exerted on the bladder wall. Tissue deformations in response to this body force launches an anti-symmetric Lamb wave that propagates in two opposite directions along the bladder wall (Fig 1). Given a wall thickness of 2*h*, wave dispersion can be described using the following characteristic equation

$$4k_{L}^{3}\beta \text{tanh}(\beta h)=k_{s}^{4}-\beta^{2}\text{tanh}(k_{L}h)$$ (1)

where $\beta =\sqrt{k_{L}^{2}-k_{s}^{2}}$, *k*_*L*_ = ω/*c*_*p*_ is the Lamb wave number, ω is the angular frequency, *c*_*p*_ is the Lamb wave phase velocity, $k_{s}=\omega ⁄\sqrt{\rho /\mu }$ is the shear wave number, *ρ* is the tissue density, *μ* is the complex shear modulus (*μ* = *μ*_1_ + *iμ*_2_) [19].

High speed imaging of the tissue vibrations in response to radiation force enables tracking of the particle displacement from which group velocity and phase velocity dispersion can be obtained [19]. The analytic model presented in Eq 1 is used to find the closest fit that describes the experimental dispersion data through an exhaustive search. The complex modulus from this optimal solution is reported as the viscoelastic parameters of the bladder wall. Only modulus of elasticity is considered in this study represented by *μ* hereafter.

### Urodynamic study

Standard UDS consists of bladder catheterization through the urethra for gradual filling and simultaneous measurement of the intravesical pressure by a pressure sensor. An additional sensor is used to measure the pressure outside the bladder (i.e., abdominal pressure) (Fig 1). The net detrusor pressure on the bladder wall is then calculated as the difference of the two pressure values. A schematic of the concurrent UBV-UDS setup is shown in Fig 1. Pressure values, pump volume, filling rate and other parameters are recorded in a standard UDS chart to be reviewed by the physician after completion of the study. Prior to each study, patient’s bladder is emptied and pressure sensors are placed and calibrated to ensure consistent readings. A computerized pump then starts inserting fluid into the bladder following standard clinical guidelines [8]. In this study, the compliance was calculated based on the guidelines described in [8] where the bladder with compliance > 40ml/H_2_O was considered compliant and the bladder with compliance < 40ml/cmH_2_O was considered as noncompliant.

### Patient population

Adult patients with neurogenic bladder referring to Mayo Clinic urology department for routine UDS were recruited for UBV study. The study was approved by the Mayo Clinic institutional review board (IRB), and written consent was obtained from patients prior to the examination.

### UBV data collection and processing

A programmable ultrasound machine, Verasonics system (Verasonics, Redmond, WA) equipped with a curved linear array (C4-2, ATL/Philips, Bothell, WA) with center frequency of 2.5 MHz was used. UDS bladder filling was performed at 50ml increments and at each volume two UBV measurements were performed. The filling rate was set based on standard clinical procedure guidelines [8] that recommends a filling rate of 10–100 ml/min. Along with each acquisition, a time stamp was placed on the UDS data collection chart to indicate the simultaneous detrusor pressure reading. For each UBV acquisition, an ultrasound tone burst of 600–900 μs focused on the bladder wall was transmitted. The radiation force from this tone burst resulted in Lamb waves along the bladder wall. The propagation of these waves was then tracked at 2500 frames per second using ultrasound plane-wave imaging with three angles of compounding [20]. Using recorded in-phase and quadrature (IQ) data, particle displacement was calculated along the wall trajectory using the autocorrelation technique in [21]. Spatial-temporal maps of the wave propagation were then used for both group velocity calculation as well as dispersion analysis for viscoelasticity parameter estimation using the wave characteristics in Eq 1. Only modulus of shear elasticity was used in this study represented as *μ*.

### Lamb wave excitation, imaging and elasticity estimation

**Fig 2:**
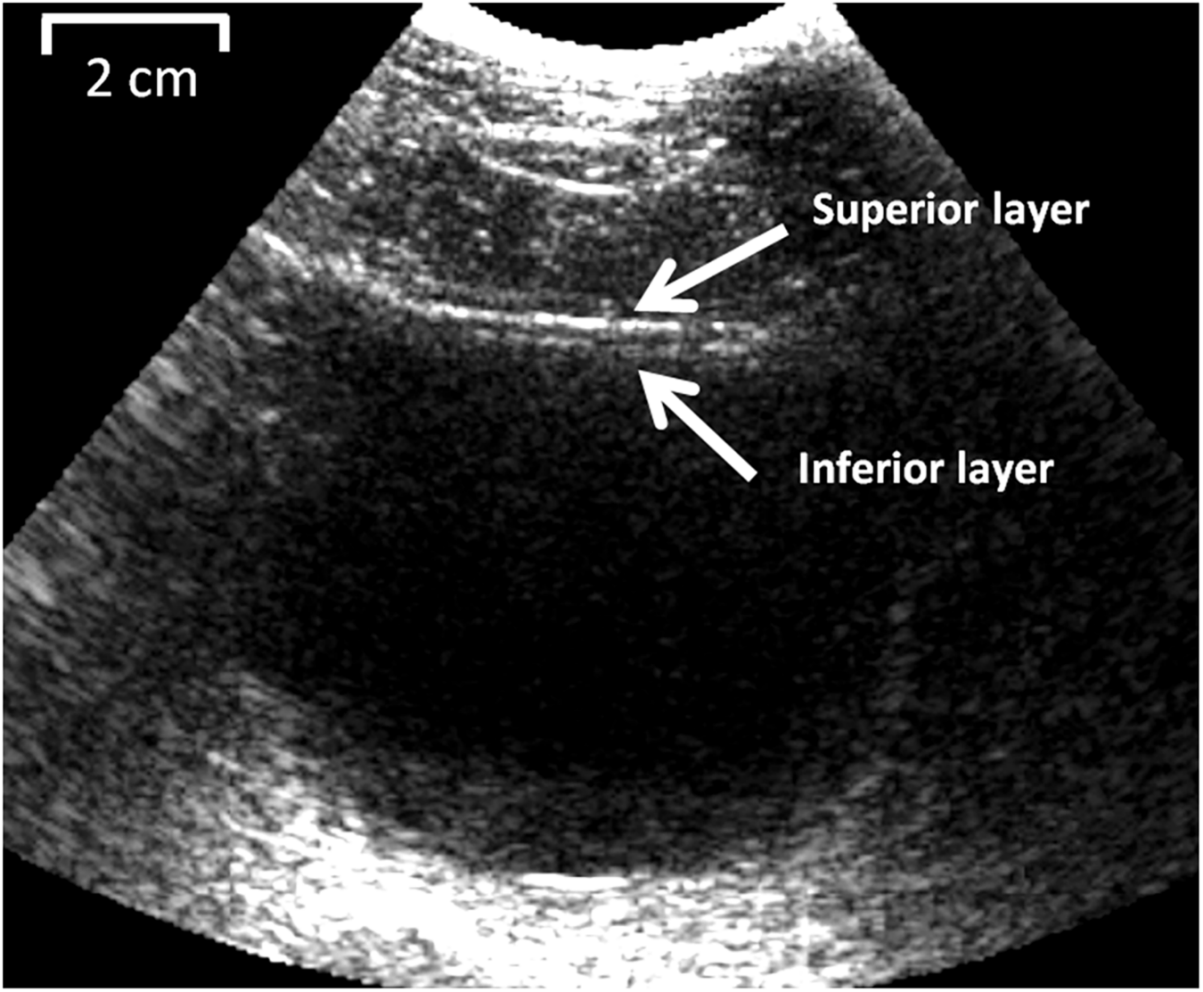
Bladder cavity and detrusor layer (inferior layer) as seen in the B-mode ultrasound image.

Fig 2 shows the B-mode image of a patient’s bladder undergoing UBV and UDS. The large hypoechoic region corresponds to the bladder intravesical area filled with saline during UDS study. Both superior and inferior sides of the bladder wall are highlighted which define the wall thickess. This thickness is used in the Lamb wave model for inversion of the wave equations (Eq 1).

**Fig 3:**
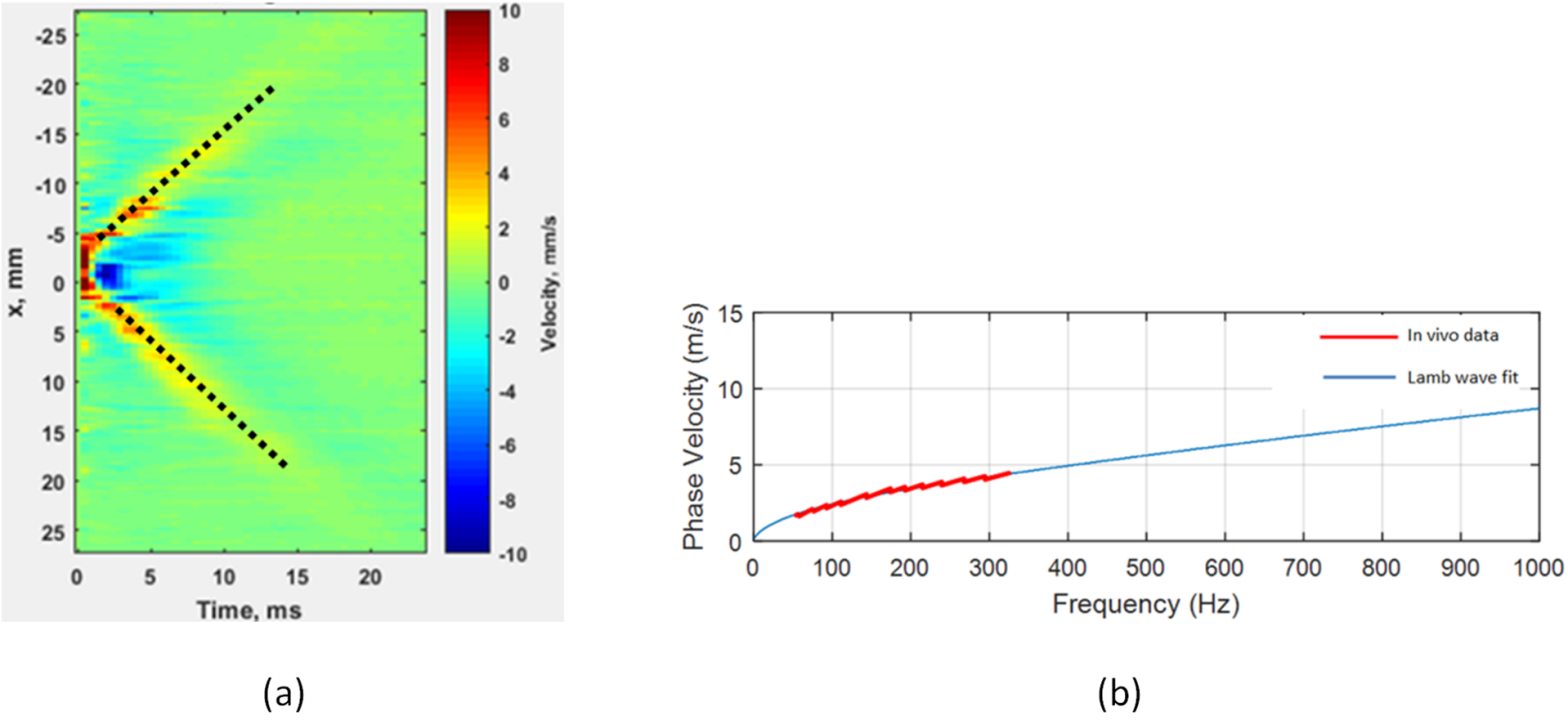
(a) Spatial-temporal map of the bladder wall motion in response to acoustic radiation force. Dotted lines represent the least square regression for group velocity calculation. The color shows the amplitude of the particle velocity in mm/s. (b) Phase velocity dispersion curve from the two-dimensional Fourier analysis of the displacement data and Lamb wave fit for calculation of the elasticity.

Fig 3(A) shows the spatial-temporal map of the paritcle motion velocity due to radiation force excitation. The color in this map repesents the tissue particle velocity in mm/s. The resulted Lamb wave porpagates in two opposite direcions (left and right) at a speed which is dependent on the bladder wall viscoelastic properties and its thickness at each volume. Least square regeression of the wave peak amplitude tracking, also overlaid on the maps, are used for group velocity calculation. The average of wave speeds in two directions is reported as the group velocity.

Fig 3(B) represents the phase velocity dispersion acquired from the spatial-temporal data along the bladder wall. This curve is used for inversion of the wave equations [19] and extracting the shear modulus of elasticity, *μ*.

### Statistical analysis

For each patient, at each incremental filling volume, bladder wall mechanical properties in terms of Lamb wave group velocity squared and shear elasticity were calculated using UBV method. At each cystometric volume, the corresponding detrusor pressure reading was collected from the UDS. Wall mechanical properties were then correlated with the UDS detrusor pressure data using Pearson correlation. A student t-test was used to analyze the significance of correlations between UBV parameters and UDS detrusor pressure. All statistical analyses were performed in MedCalc ver. 15.8 (MedCalc, Seoul, Republic of Korea).

## Results

Seventy patients, 56 male and 14 female, were included in this study. Mean age was 52.19±15.9 years and mean body mass index (BMI) was 25.91±4.32.

### UBV-UDS measurement

**Fig 4:**
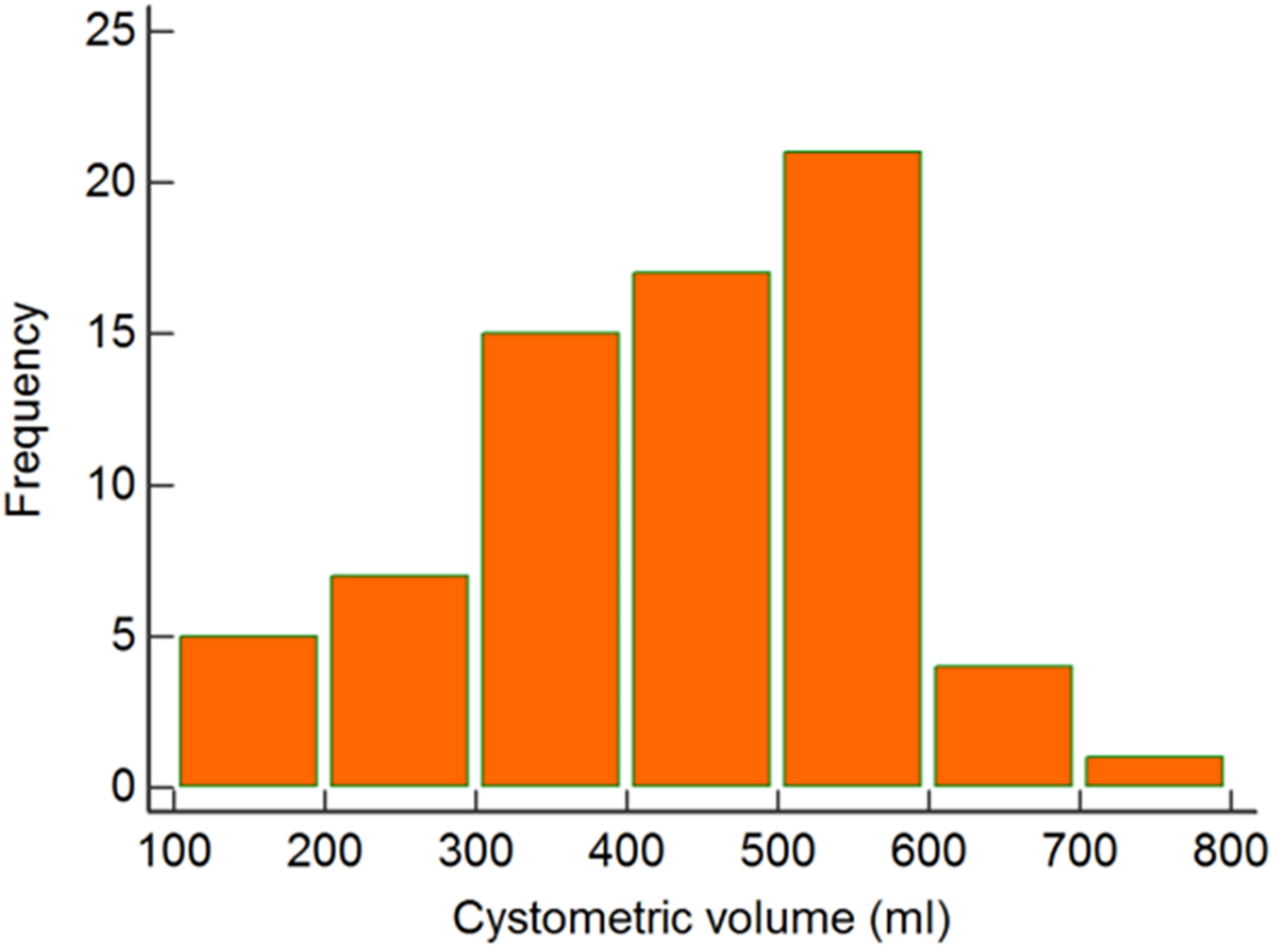
(a) Distribution of cystometric volume in 70 patients.

The median cystometry filling volume was 438.5ml (CI: 398.66–486.81) and mean was 415.93ml (CI: 387.28–444.57). The study ended at 500ml for 19 (27%) patients and 6 (8%) patients could not maintain more than 250ml due to early leaking. Fig 4 depicts the distribution of the cystometric volumes in all patients.

**Fig 5:**
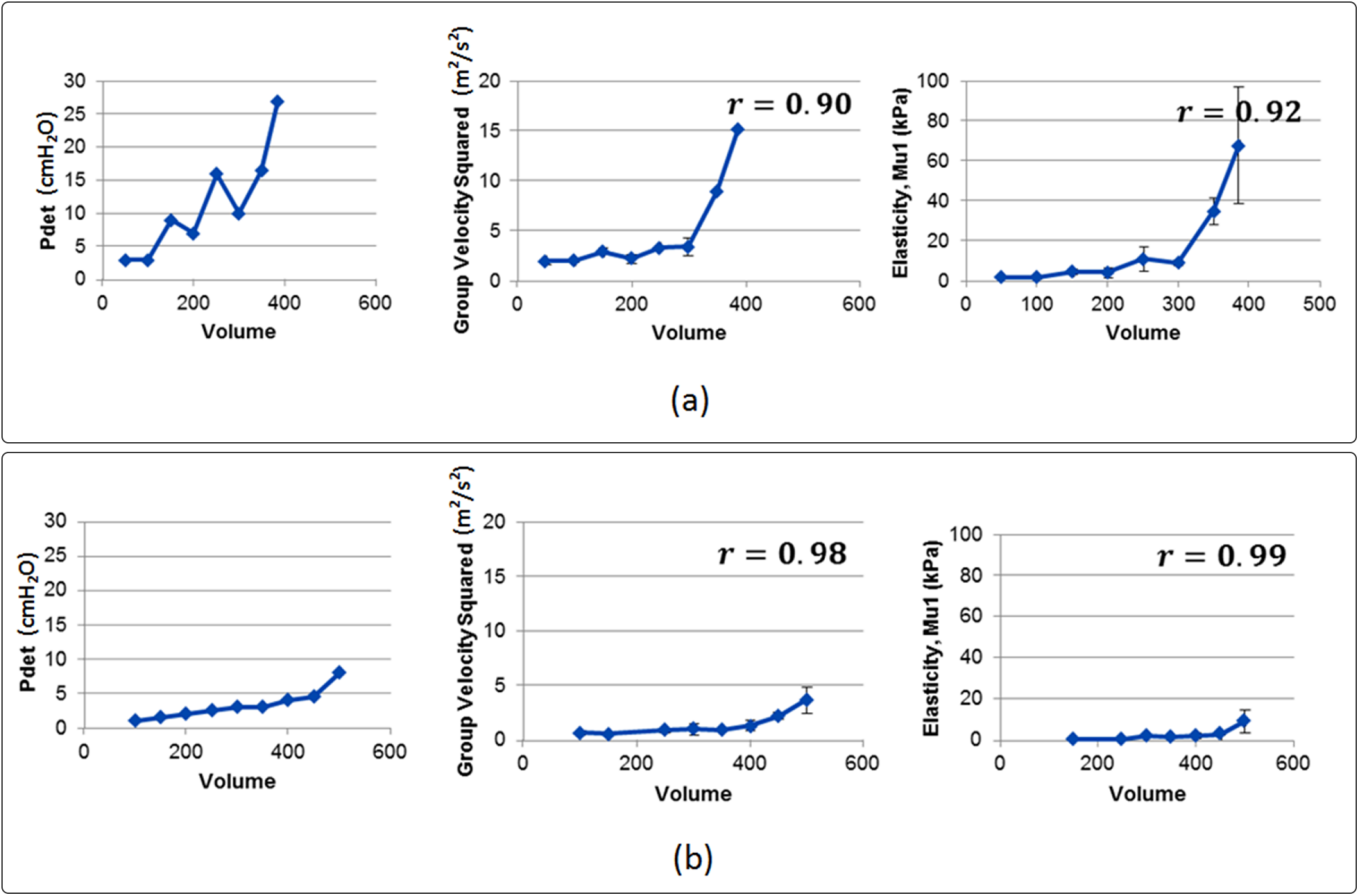
(a) From left to right: detrusor pressure, estimated group velocity squared and shear elasticty in a noncompliant patient (compliance < 18.75). (b) from left to right: detrusor pressure, estimated group velocity squared and shear elasticity in a compliant patient (compliance > 62.5)

Fig 5 shows the UDS and UBV measurements in two patients. Fig 5(A) presents the UDS-UBV measurments in a noncompliant bladder (compliance < 18.75) where a rapid change in detrusor pressure is seen at low volumes. The same trend can be obsered in the estimated group velcity squared and shear elasticity with UBV-UDS Pearson correlation values of 0.90 and 0.92, respectivly. Fig 5(B) presents the UDS-UBV measurments in a compliant bladder (compliance > 62.5). Both UDS and UBV parameters show a smooth increasing trend with increasing volume which is indicative of a compliant bladder. Pearson correlation of 0.98 and 0.99 were observed between detrusor pressure, *Pdet*, and group velocity squared and shear elasticity, repsectively.

### Statistical analysis results

**Fig 6:**
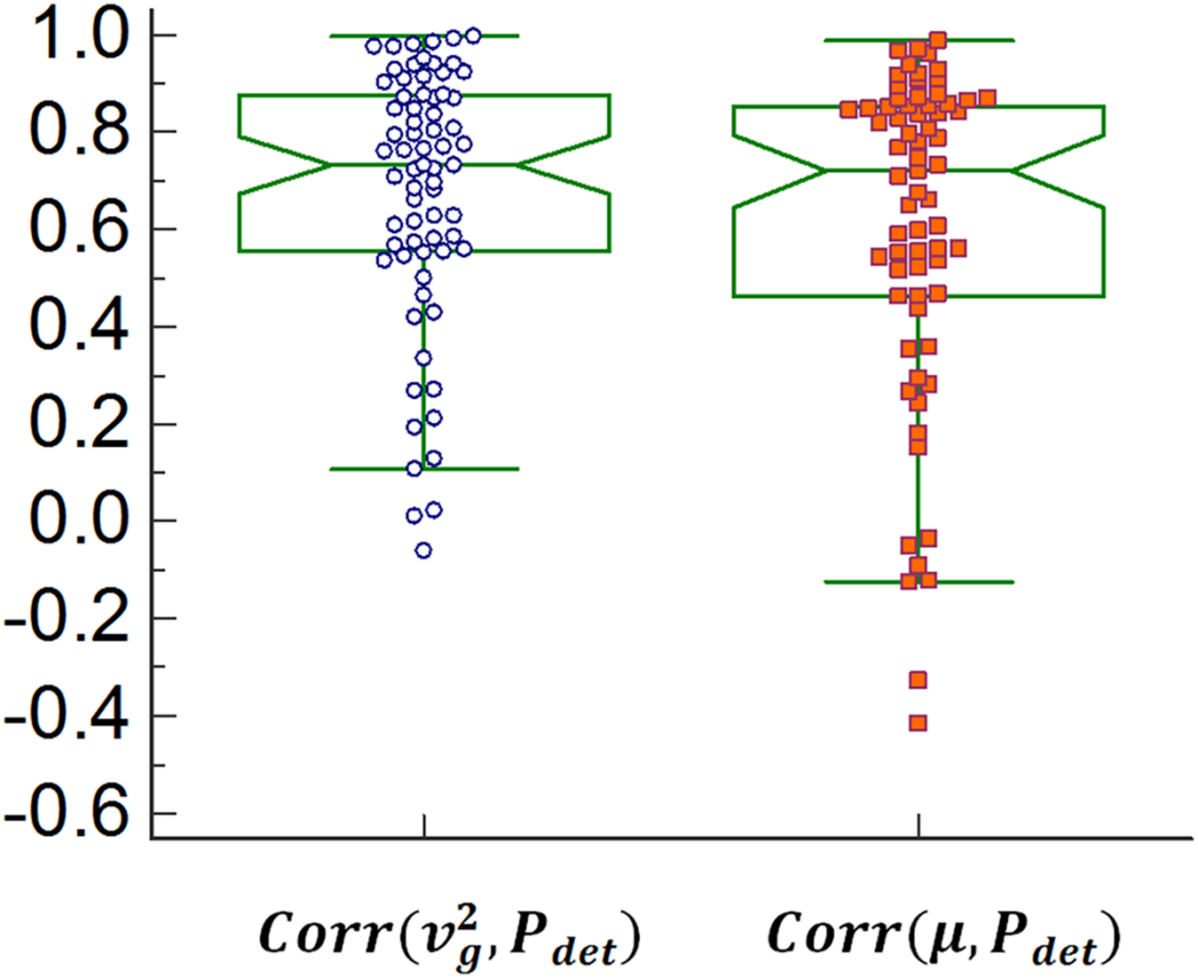
Distribution of Pearson correlation between different parameters of UBV and UDS detrusor pressure (*Pdet*). For each group the width of the notches show the 95% confidence interval and data points with different markers present outliers in each group.

Fig 6 shows the notch plot of the Pearson correlation values between UBV group velocity squared and UDS detrusor pressure, $Corr(v_{g}^{2},P_{det})$, and shear modulus and UDS detrusor pressure *Corr*(*μ*, ***P***_***det***_) acquired from 70 patients. A summary of the statistical analysis of correlation values between UBV parameters and UDS detrusor pressure is presented in Table 1.

**Table 1:** Summary of the statistical analysis of correlation between UBV and UDS.

	$Corr(v_{g}^{2},P_{det})$	*Corr*(*μ*, *P*_*det*_)
Median Pearson correlation (95% CI)	0.73 (0.64–0.80)	0.72 (0.56–0.82)
Mean Pearson correlation (95% CI)	0.67 (0.61–0.74)	0.60 (0.52–0.69)
t-test value = 0 correlation	P < 0.0001*	P < 0.0001*
t-test value = 0.5 correlation	P < 0.0001*	P = 0.0127*

* p-value < 0.05 was considered significant.

## Discussion

In this study we examined the correlation of bladder wall mechanical properties measured by ultrasound bladder vibrometry with UDS detrusor pressures measured at different cystometric volumes. The first step in UBV is to find the bladder wall using conventional B-mode ultrasound imaging. Our study showed that in most cases, bladder wall can be easily detected on the ultrasound images, even at low filling volumes. Using acoustic radiation force-based excitation followed by high frame rate imaging we were able to excite transient waves and track both their group velocity and phase velocity. The latter was used for a dispersion analysis from which elasticity was acquired. Our results showed strong correlation between group velocity squared and elasticity with detrusor pressure where median correlations were higher than 0.70. It was further observed that, group velocity squared and elasticity present similar correlations with detrusor pressure, with more statistical dispersions observed in the elasticity correlation data compared to the group velocity squared (Fig 6). This can be related to the Lamb wave model inversion that might results in erroneous model parameters when radiation force displacement data are not of high quality. A factor that can affect the quality of Lamb wave excitation and measurement is patient’s BMI. Such effect has been also observed in the study of liver fibrosis staging using shear wave elastography [22]. Increased BMI causes a faster decay in the energy of compressional waves which create the acoustic radiation force. Additionally, excessive abdominal fat can create strong imaging clutters which in turn can reduce the accuracy in estimation of the balder wall thickness. While reduced radiation force can affect the estimation of both UBV group velocity squared and elasticity, erroneous wall thickness estimation only impacts the quality of the inversion procedure required for measurement of the elasticity. Hence, compared to elasticity, group velocity squared provided better mean and median correlation with detrusor pressure, assuring the existence of a reliable surrogate for UBV shear elasticity in challenging cases. A student t-test showed that the correlation between UBV and UDS was significantly higher than 0.5 for both UBV parameters.

One limitation of our study was that UBV measurements were acquired only at incremental volume points rather than continuously. Hence, short-term changes in bladder physiological or neurological state, such as contractions and overactivity, might not be adequately captured using this setup. A more versatile implementation of UBV with the capability of continuously monitoring the bladder wall with filling volume is a future aim as an extension of the current method to address this issue. Moreover, continuous monitoring of elasticity would enable time-dependent analysis of the bladder’s physiological and neurological state via analysis of the elasticity parameters in form of linear and nonlinear time series similar to methods in [23, 24].

The overall results of this study prove the suitability of UBV as an alternative technique for fast an accurate assessment of the bladder physiological and neurological conditions in terms of mechanical compliance. This method is especially attractive for patients who frequently require undergoing UDS due to a high risk of rapid bladder deterioration or due to closer monitoring purposes in response to different medications. UDS requires catheterization for both filling the bladder and measuring the detrusor pressure. UBV parameters, on the other hand, can be obtained noninvasively. Furthermore, for patients who have the capability to void voluntarily, UBV can be conducted through natural filling of the bladder and fractional voiding. This method has shown to be feasible in a previous study [19] on healthy volunteers thus, in the future, UBV could be considered as an alternative for UDS.

### Conclusions

The results of this study show that UBV parameters were significantly correlated with UDS measurements of the detrusor pressure, hence UBV may serve as a surrogate for accurate evaluation of the bladder detrusor state in a noninvasive fashion. The entire procedure is based on ultrasound which can be easily translated to the clinical setup using currently available ultrasound machines. This new technique can provide the flexibility to assess the bladder detrusor state in shorter time intervals with lower cost and ease of operation.
